# Multi-tyrosine kinase inhibitors in preclinical studies for pediatric CNS AT/RT: Evidence for synergy with Topoisomerase-I inhibition

**DOI:** 10.1186/1475-2867-11-44

**Published:** 2011-12-29

**Authors:** Aarthi Jayanthan, Delphine Bernoux, Pinaki Bose, Karl Riabowol, Aru Narendran

**Affiliations:** 1 Laboratory for Pre-clinical and Drug Discovery Studies, Pediatric Oncology Experimental Therapeutics Investigators Consortium (POETIC) and Division of Pediatric Oncology, Alberta Children's Hospital, 2888 Shaganappi Trail NW, Calgary, T3B 6A8, Canada; 2 Departments of Biochemistry and Molecular Biology and Oncology, University of Calgary, 3330 Hospital Drive NW, Calgary, T2N 4N1, Canada

## Abstract

**Background:**

Currently, Atypical Teratoid Rhabdoid Tumor (AT/RT) constitutes one of the most difficult to treat malignancies in pediatrics. Hence, new knowledge of potential targets for therapeutics and the development of novel treatment approaches are urgently needed. We have evaluated the presence of cytokine pathways and the effects of two clinically available multi-tyrosine kinase inhibitors for cytotoxicity, target modulation and drug combinability against AT/RT cell lines.

**Results:**

AT/RT cell lines expressed measurable quantities of VEGF, FGF, PDGF and SDF-1, although the absolute amounts varied between the cell lines. The targeted receptor tyrosine kinase inhibitor sorafenib inhibited the key signaling molecule Erk, which was activated following the addition of own conditioned media, suggesting the existence of autocrine/paracrine growth stimulatory pathways. The multi-tyrosine kinase inhibitors sorafenib and sunitinib also showed significant growth inhibition of AT/RT cells and their activity was enhanced by combination with the topoisomerase inhibitor, irinotecan. The loss of cytoplasmic NF-kappa-B in response to irinotecan was diminished by sorafenib, providing evidence for a possible benefit for this drug combination.

**Conclusions:**

In addition to previously described involvement of insulin like growth factor (IGF) family of cytokines, a multitude of other growth factors may contribute to the growth and survival of AT/RT cells. However, consistent with the heterogeneous nature of this tumor, quantitative and qualitative differences may exist among different tumor samples. Multi-tyrosine kinase inhibitors appear to have effective antitumor activity against all cell lines studied. In addition, the target modulation studies and drug combinability data provide the groundwork for additional studies and support the evaluation of these agents in future treatment protocols.

## Background

Atypical Teratoid Rhabdoid Tumor of the central nervous system (CNS AT/RT) is a highly malignant neoplasm of infants and young children. A biallelic inactivation of the *hSnf5/Ini1 *gene located in 22q11.2 is a characteristic molecular defect in these tumors [1-4]. Murine knock-out models have confirmed that hSnf5*/Ini1 *is a tumor-suppressor gene [5], but the details of its exact role in the initiation and growth of the AT/RT are still being investigated. To date, studies showing INI1 interaction with key signaling molecules suggest its potential to modify the response to factors that mediate cell growth and differentiation programs [6-8]. There is emerging evidence for the existence of autocrine and/or paracrine growth factor signaling pathways in these cells. Previously, we were able to maintain disseminated AT/RT cells in culture by the addition of autologous CSF to culture medium [9]. Agents that inhibit IGF-IR activity have been shown to diminish tumor cell growth and targeting of IGF-IR expression with antisense oligonucleotides resulted in increased apoptosis and sensitivity to a number of chemotherapeutic agents [10]. Furthermore, Arcaro and colleagues have shown evidence for autocrine signaling by insulin and its receptor (IR) in AT/RT cells, which involves the PI3K/Akt pathway [11]. These findings suggest that abnormally regulated cytokine pathways and their downstream signaling molecules can be effective targets for therapeutics in AT/RT.

Ultra structurally, AT/RT often presents as a polymorphous tumor with overlapping morphologic features consisting of primitive neuroectodermal tumor (PNET), mesenchymal, rhabdoid and epithelial components. This phenotypic heterogeneity is likely to be aided by multi-level cross stimulation of growth and survival pathways and signaling molecules. As such, a single-targeted agent may not be the optimal choice, as these agents may permit the development of salvage or escape mechanisms. However, by virtue of their ability to interfere with a diverse array of signaling molecules, including cytokine receptor kinases, multi-targeted inhibitors may provide a therapeutic advantage in the treatment of AT/RT. In the recent past, tyrosine kinase inhibitors with multiple targets have been found to have clinically achievable activity and acceptable tolerability in studies against heterogeneous malignancies [12]. In this study, we have evaluated two such agents, sunitinib and sorafenib, for *in vitro *activity and drug combinability against three AT/RT cell lines.

## Results

### Cytokine expression by AT/RT cells

Quantitative evaluation of the cytokines found in the culture supernatants of the three AT/RT cell lines was performed by multiplex assay. Data presented in Table 1 shows significant presence of six of the 65 cytokines examined. However, with the exception of VEGF, other cytokines showed quantitative variations between the cell lines. For example, in comparison to BT12 and KCCF1 cells, BT16 cells did not express measurable levels of IL-8 and MCP-1, and expressed only a very low level of SDF-1. Although BT12 supernatants contained higher quantities of all other cytokines, the level of FGF(b) was measurably lower in this sample, indicating the potential heterogeneity in the presence of different cytokines in the tumor micro-environment.

**Table 1:** Quantitative analysis of cytokines present in the spent culture supernatants of AT/RT cell lines

Cytokine (ρg/ml)	FGF(b)	IL-8	MCP-1	PDGF-AA	VEGF	SDF-1
Media Control	UD	UD	UD	UD	16.26	UD

BT 12 Supernatant	14.93	144.7	63.38	157.76	226.67	228.28

BT 16 Supernatant	82.45	UD	UD	197.06	261.04	4.74

KCCF1 Supernatant	40.35	10.18	UD	43.35	124.57	190.17

UD - undetectable

### Sensitivity of AT/RT cell lines to multi-targeted tyrosine kinase inhibitors and irinotecan

**Figure 1:**
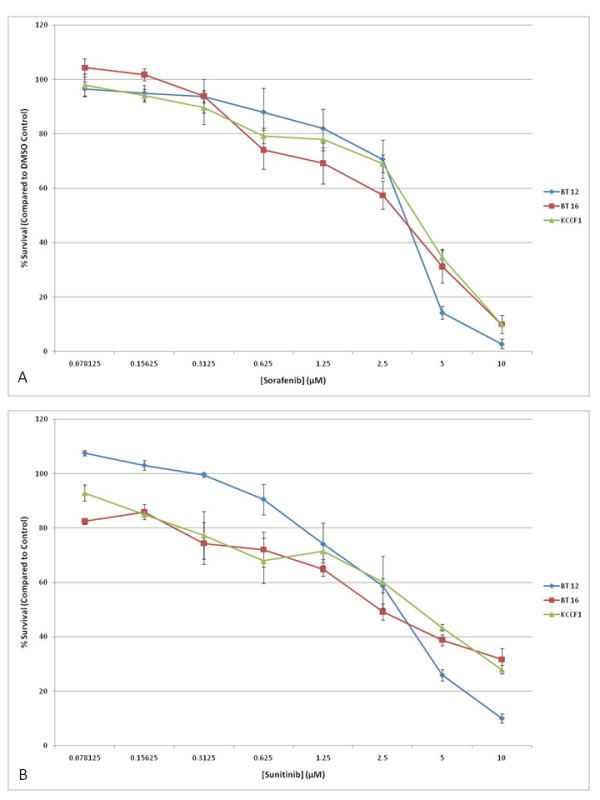
**Sorafenib and sunitinib induce cell growth inhibition in AT/RT cell lines *in vitro***. Triplicate cultures of three AT/RT cell lines were incubated with increasing concentrations of sorafenib (A) or sunitinib (B). Appropriate dilutions of DMSO were also included as vehicle control. After four days in culture, cell viability was measured and plotted as a percentage of cell survival compared to equivalent vehicle control. Bars indicate SD. Data presented above are representative of five separate experiments. IC_50 _values calculated from these data are given in Table 2.

**Figure 2:**
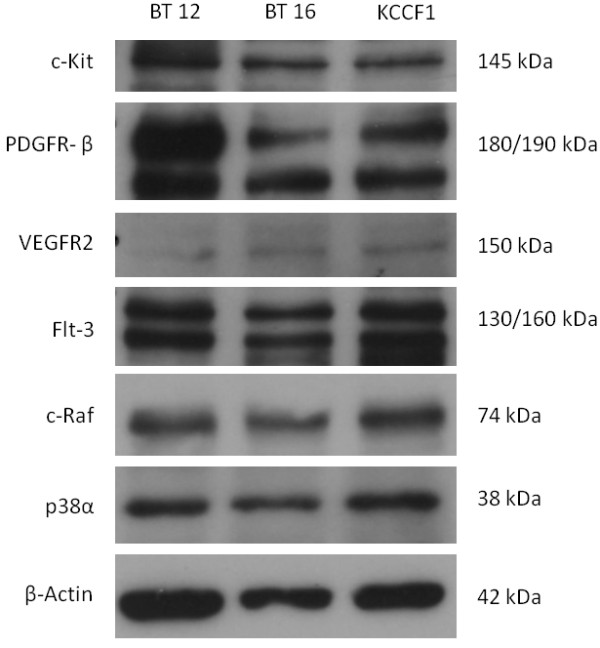
**Expression of targets of sorafenib and sunitinib in AT/RT cell lines**. Using Western blot analysis, it was determined that AT/RT cell lines express receptor tyrosine kinase targets of sorafenib and sunitinib: c-Kit, PDGF-Rβ, VEGFR2 and Flt-3. In addition, these cell lines express intracellular targets of sorafenib: c-Raf and p38α.

The presence of a multitude of cytokines in the culture supernatants of the AT/RT cell lines indicated the potential for autocrine or paracrine growth sustaining processes utilizing these molecules. Therefore, we wanted to investigate the effects of agents that have been shown to interfere with the activity of such receptor pathways. Sorafenib and sunitinib have been shown to inhibit the activity of a number of cytokine receptors, including vascular endothelial growth factor receptor (VEGFR), platelet-derived growth factor receptor (PDGFR), stem cell factor receptor (c-Kit) and FMS-like tyrosine kinase-3 (Flt-3) [13,14]. In the next set of experiments, the three AT/RT cell lines were evaluated for sensitivity to sorafenib and sunitinib by *in vitro *cytotoxicity assays. Figures 1A and 1B show the dose dependent inhibition of AT/RT cell growth by these agents. From these data, IC_50 _values were calculated and presented in Table 2. IC_50 _values for each cell line ranged from 2.8 to 3.6 µM for sorafenib and 3.2 to 3.7 µM for sunitinib. As a means to further support the targeted inhibition of receptor pathways by sorafenib and sunitinib, the expression of proteins targeted by these inhibitors was determined by Western blot analysis. It was found that all three AT/RT cell lines expressed receptor tyrosine kinases c-Kit, PDGF-Rβ, VEGFR2 and Flt-3, as well as the intracellular targets of sorafenib, c-Raf and p38α (Figure 2).

**Table 2:** *In vitro *cytotoxicity of sorafenib, sunitinib, irinotecan and SN-38 against three AT/RT cell lines

Inhibitor, IC_50 _(μM)	BT 12	BT 16	KCCF1
**Sorafenib**	3.3	2.8	3.6

**Sunitinib**	3.2	3.2	3.7

**Irinotecan**	2.0	4.1	6.7

**SN-38**	0.09	0.03	4.6

IC_50 _- half maximal inhibitory concentration

### Synergistic activity of irinotecan with sorafenib and sunitinib

**Figure 3:**
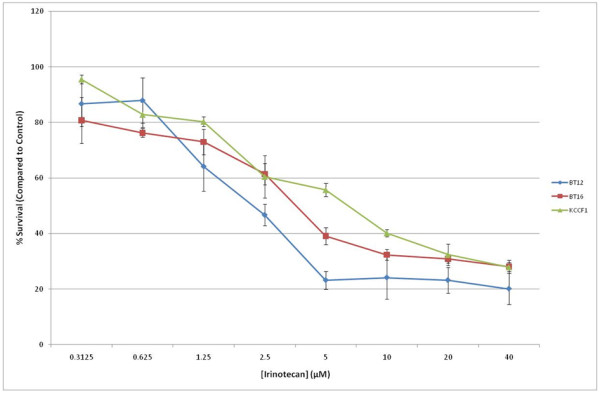
**Irinotecan inhibits the in vitro growth of AT/RT cells**. Triplicate cultures of AT/RT cell lines were cultured in the presence of increasing concentrations of irinotecan. Following four days in culture, cell growth inhibition was measured by Alamar blue assay. Percentage of cell survival was calculated by comparison of treated cells to control cells. Similar results were obtained in five separate experiments. IC_50 _values obtained from these data are given in Table 2.

Previous studies have indicated the potential activity of the new generation topoisomerase I inhibitor irinotecan against brain tumors and its ability to increase the activity of agents that block VEGF activity [15]. To deduce the role of irinotecan in potential combination therapies, we first analyzed its activity as a single agent. Figure 3 shows the cytotoxic effects of irinotecan against the three AT/RT cell lines. The IC_50 _values (Table 2) ranged from 2.0 to 6.7 μM, with BT12 cell line exhibiting a significantly lower IC50 value of 2 μM compared to other two cell lines. We also tested the activity of the active metabolite of irinotecan, SN-38 against the three AT/RT cell lines. The IC_50 _values (Table 2) ranged from 0.03 to 4.6 μM, with KCCF1 exhibiting a significantly higher IC_50 _value compared to the other two cell lines. Next, we evaluated the drug combinability of irinotecan with sorafenib and sunitinib. The ability of a fixed concentration of irinotecan (IC_25_) to reduce the IC_50 _values in serially diluted sorafenib and sunitinib was evaluated by *in vitro *cytotoxicity assays and combination indices (CI) were calculated according to the method of Chou and Talalay [16]. Table 3 shows the respective CI calculations. Combination index values less than 1 (CI < 1) indicates synergy between two agents.

**Table 3:** Combination indices in drug combination studies of irinotecan and the multi-kinase inhibitors sorafenib and sunitinib

Inhibitor, CI	BT 12	BT 16	KCCF1
**Sorafenib**	0.7	0.8	0.9

**Sunitinib**	0.9	0.3	0.4

### Modulation of intracellular signaling molecules by sorafenib

**Figure 4:**
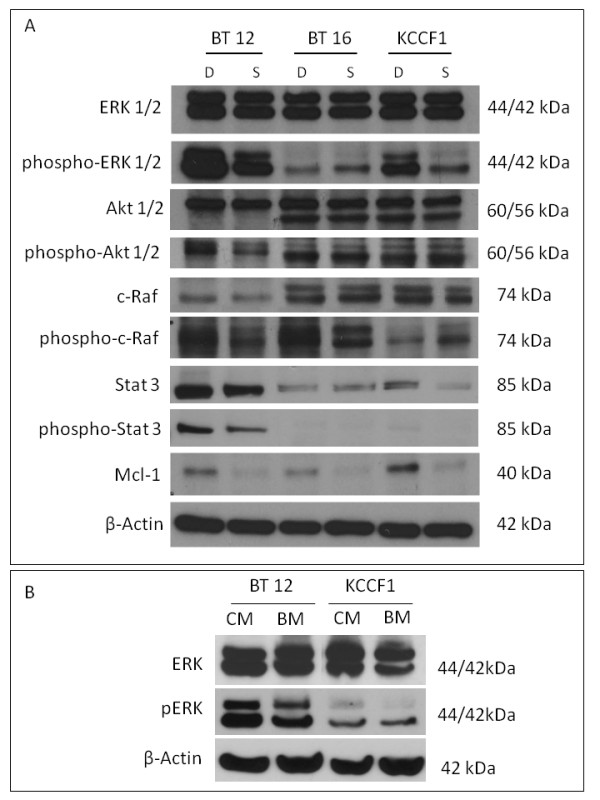
**The multi-kinase inhibitor sorafenib induces alterations in cellular signaling and apoptosis regulators in AT/RT cells in response to conditioned medium**. Addition of autologous conditioned medium to briefly serum starved cells provides an avenue to evaluate the potential responses that may occur during autocrine/paracrine growth stimulation. In the presence of sorafenib (S), the conditioned medium induced reduced phosphorylation of Erk1/2 (BT12, KCCF1), Akt1/2 (BT12), c-Raf (BT12), Stat3 (BT12, KCCF1) compared to DMSO (D) control. Under these conditions, an increase in phosphorylation of c-Raf was noted in KCCF1 cells. However, the pro-survival protein Mcl-1 was suppressed by sorafenib in all three cell lines (A). Compared to basal medium (BM), conditioned medium (CM) induced phosphorylation of signaling regulators, such as Erk (B). The data shown are typical of three separate experiments.

Our initial set of experiments involved the screening of changes in the activation status of signaling molecules in response to treatment with sorafenib in AT/RT cells. Exponentially growing cells were treated with 10 µM of sorafenib, or appropriate vehicle control, and cell lysates were analyzed by Western blots as described in materials and methods. Data presented in Figure 4A shows that, in most cases, sorafenib decreased the levels of multiple signaling components in AT/RT cells Significant loss of phosphorylated cell growth regulators was seen in all AT/RT cells although variations were seen among the different cell lines; Erk1/2 (BT12, KCCF1), Akt 1/2 (BT12), c-Raf (BT12, BT16) and Stat3 (BT12, KCCF1). Loss of the cell survival molecule Mcl-1, however, was found in all three cell lines studied.

The addition of conditioned medium to cells that are serum starved provides an experimental model to study the autocrine/paracrine pathways mediated by secreted cytokines. Agents that block such activation pathways may contribute to ultimate growth inhibitory activities and provide a rationale for investigating receptor tyrosine kinase inhibitors as targeted therapeutics. In the next set of experiment we demonstrate that indeed the conditioned media from AT/RT cells induce Erk phosphorylation, which has been shown to be one of the downstream targets of sorafenib activity (Figure 4B).

**Figure 5:**
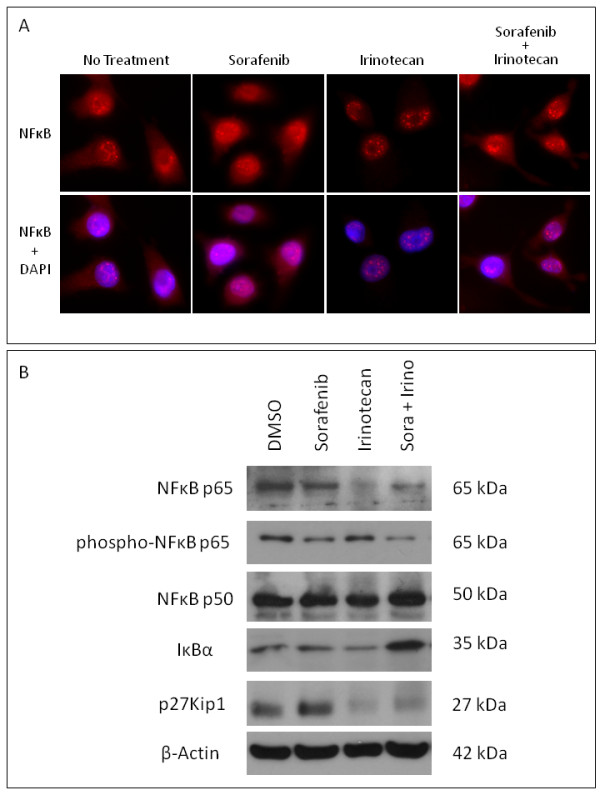
**Cytoplasmic NF-*κ*B levels are lowered by irinotecan but sorafenib reduces this effect**. BT12 cells were incubated with sorafenib or vehicle for 30 minutes followed by treatment with irinotecan for an additional 2 hours. For indirect immunofluorescence (A), cells were fixed and stained with antibodies to NF-*κ*B followed by fluorescent labeled secondary antibodies. Concurrent DAPI stain was used to localize the nuclei (lower panel). Slides were visualized using a fluorescent microscope and random fields were photographed. The cytoplasmic staining seen in untreated and sorafenib treated cells was significantly reduced following treatment with irinotecan. However, the addition of sorafenib enabled the cells to maintain cytoplasmic staining in the presence of irinotecan. For Western blot analysis (B), cytoplasmic proteins were analyzed using antibodies against NF*κ*B p65 and p50, phospho-NF*κ*B p65, I*κ*Bα and p27Kip1. In the presence of irinotecan, there was a loss of cytoplasmic NF-*κ*Bp65, but in the presence of sorafenib, this loss was greatly reduced, corresponding to a decrease in phosphorylation of NF-*κ*Bp65. In addition, compared to treatment with sorafenib or irinotecan alone, there was increased expression of I*κ*Bα following treatment with sorafenib and irinotecan. Lastly, following treatment with irinotecan and sorafenib irinotecan combination, there was decreased expression of p27Kip1 compared to sorafenib treatment alone.

Previous studies have suggested that the activation of NF-kappa-B (NF-*κ*B) in response to chemotherapeutic agents, including irinotecan may relate to the generation of resistance in cancer cells [17,18]. To further evaluate the input of MTK inhibition in this process, we evaluated the effect on NF-*κ*B in response to irinotecan as a single agent and then in combination with sorafenib. Using BT12 cells, we examined the presence of cytoplasmic NF-*κ*B by indirect immunofluorescence. Cells receiving sorafenib, irinotecan or the combination were fixed and stained with antibodies to NF-*κ*B. The slides were visualized under a fluorescent microscope and random fields were photographed. Representative photographs in Figure 5A show that the cytoplasmic staining of NF-*κ*B is unchanged in sorafenib treated cells compared to control cells, but there is a significant reduction in such staining when the cells were treated with irinotecan. However, this loss is reduced by combination with sorafenib. As activated NK-*κ*B translocates from cytoplasm during the activation process, this indicates that irinotecan and sorafenib combination leads to potentially reduced translocation of NF-*κ*B compared to irinotecan alone. To further confirm this possibility, cytoplasmic extracts of cells treated with either irinotecan alone or the irinotecan and sorafenib combination were evaluated by Western blot analysis. Results presented in Figure 5B show that NF-*κ*B p65 detected with irinotecan alone is significantly less compared to levels detected when the cells received added sorafenib. This suggests that sorafenib may be able to reduce the translocation and hence the activation on NF-*κ*B that follows irinotecan treatment. In addition, compared to treatment with sorafenib or irinotecan alone, the cells treated with the combination showed increased I*κ*Bα, providing further evidence for stabilization of NF-*κ*B under this condition.

Previous studies have shown that the tumor suppressor gene CDKN1B encodes for a 27-kDa cyclin-dependent kinase inhibitory protein, p27Kip1, which inhibits cell proliferation and motility [19]. Our initial screening studies (data not shown) have shown that AT/RT cells also down regulate p27Kip1 in response to irinotecan. Sorafenib, however, did not have this effect and the irinotecan sorafenib combination did not lead to additional loss of p27Kip1 (Figure 5B).

## Discussion

Currently, the prognosis for children with AT/RT is very poor. Occasional anecdotal reports of successful treatment are noted; but optimal therapy or even effective therapy has not been achieved in most cases. The chemotherapeutic agents classically used are cyclophosphamide, cisplatin, etoposide, vincristine, carboplatin and ifosfamide [1]. The setback is that tumors seem to be responsive initially but develop resistance [20]. However, recent evidence suggests that improved survival can be achieved with the use of more aggressive treatment approaches, including dose-intense chemotherapy and adjuvant radiation therapy [21-23]. It has also been shown that radiotherapy is crucial to improve the survival rate of children with AT/RT [24,25]. Chi and colleagues have described an innovative treatment approach consisting of an aggressive multimodality approach [26]. This protocol is the first prospective investigation consisting of surgery, radiation therapy combined with multi-agent systemic and IT chemotherapy and has resulted in a significant improvement in time to progression and overall survival of AT/RT patients. In general, the striking potential for long-term consequences of treatments that include radiation in these very young children necessitates trials with new therapeutics and treatment regimens.

The role of cytokine receptor mediated growth and survival signals in rhabdoid tumors has been investigated by a number of laboratories. In addition to the effects of IGF-I described previously [27], our studies have shown the expression of significant quantities of VEGF and PDGF by all three cell lines (Table 1). Based on this, we have explored the effects of two multi-kinase inhibitors that have been shown to inhibit growth stimulatory pathways mediated by the receptors of these cytokines. Sorafenib and sunitinib are two oral multi-targeted receptor-tyrosine kinase inhibitors that are currently in clinical trials for various malignancies. Sorafenib is a multi-kinase inhibitor that inhibits the activity of c-Raf, b-Raf, vascular endothelial growth factor receptor family (VEGFR-2 and VEGFR-3), platelet-derived growth factor receptor family (PDGFR-β ) and stem cell factor receptor (c-Kit). Sunitinib is a multitargeted inhibitor of VEGFR, PDGFR-α and -β, c-Kit and Flt-3. These two agents offer broad anti-tumor efficacy through their ability to directly and indirectly inhibit these targets in concert to ultimately interfere with tumor growth, survival, and angiogenesis [28]. It has been shown in that the antiproliferative effect of sorafenib is mediated through its effect on the MAP kinase pathway (i.e., reduced Erk1/2 phosphorylation) [29]. Our studies have shown a decrease in activated Erk1/2 in two of the three cell lines (Figure 4A). In addition, we have found a decrease in the anti-apoptotic protein Mcl-1 in all three cell lines. Interestingly, the down regulation of Mcl-1 by sorafenib has been shown previously in other tumor models [30]. Mcl-1 has also been implicated in the generation of resistance to chemotherapeutic agents [31]. Though we have shown significant alterations in the activity of key signaling molecules in AT/RT cells, the contribution of off-target effects by sorafenib cannot be ruled out and awaits further analysis in biological correlative studies in xenografts and in future clinical trials of this agent.

Recently, sorafenib has been shown to inhibit proliferation and induce apoptosis in two medulloblastoma cell lines (Daoy and D283) and a primary culture (VC312) of human medulloblastoma at inhibitory concentrations very similar to that we have observed against AT/RT cells (IC_50 _approximately 2.5 µM) [32]. *In vivo *activity of sorafenib against medulloblastoma cells has also been demonstrated in a mouse xenograft model [32]. Sunitinib has been shown to induce apoptosis and growth arrest in medulloblastoma cells by inhibiting Stat3 and Akt signaling pathways [33]. In pre-clinical testing studies, Maris and co-workers have observed activity of sunitinib against rhabdoid tumor xenografts [34]. These findings support the potential of sorafenib and sunitinib as effective treatments in AT/RT. However, as a way to increase treatment efficacy and to reduce potential adverse effects of these agents, we explored additional drug combination studies.

Irinotecan has been shown to have the ability to cross the blood-brain barrier and, in preclinical investigations, has demonstrated cytotoxic activity against central nervous system tumor xenografts [35]. Recently, a Phase I trial of irinotecan by Pediatric Oncology Group (POG) was performed in children with refractory solid tumors where stable disease (4-20 cycles) was observed in seven patients with a variety of malignancies, including a patient with CNS AT/RT [36]. In recurrent malignant gliomas, combination therapy with bevacizumab and irinotecan has been shown to prolong progression-free survival in comparison with historical controls [37,38]. Our studies have also shown the ability of irinotecan to inhibit the growth of AT/RT cells and significant synergy in drug combinations involving irinotecan with either sorafenib or sunitinib (Table 3). In previous trials, despite the initial response to therapy, most patients treated with irinotecan developed resistance and showed tumor progression [39]. In the colorectal cancer model, treatment with irinotecan has been shown to lead to the activation of NF-κB. [40]. As such, the activation of the NF-κB pathway constitutes a potential mechanism of inducible resistance by malignant cells exposed to irinotecan [41]. NF-*κ*B interferes with the effect of most anti-cancer drugs through induction of anti-apoptotic genes. Targeting NF-*κ*B is therefore expected to potentiate conventional treatments in adjuvant strategies. In addition, recent studies have shown that the administration of siRNA directed against the p65 subunit of NF-κB can effectively enhance *in vitro *and *in vivo *sensitivity to chemotherapeutic agents [42]. Thus, reducing NF-κB-mediated activation may help prevent resistance potentially generated upon exposure to irinotecan. This has been confirmed in studies where a pharmacological inhibitor of the IKK2 kinase (AS602868, Serono International SA, Geneva, Switzerland) which blocks NF-*κ*B activation has been found to enhance the action of irinotecan metabolite [43]. We have explored the possibility of decreased NF-*κ*B activation as a potential mechanism in the enhanced cytotoxicity of irinotecan in the presence of sorafenib. Our studies have provided evidence for irinotecan mediated loss of cytoplasmic NF-*κ*B in AT/RT cells. However, the presence of sorafenib appears to retain NF-*κ*B in the cytoplasm as shown by Western blot analysis and indirect immunofluoresence studies. Interestingly, in Alzheimer's disease research, a similar observation was noted where the chronic treatment with sorafenib inhibited c-Raf and NF-*κ*B in the brains of the aged APPswe mice [44].

## Conclusions

In this study, we have shown that the AT/RT cell lines produce a number of cytokines and the multi-kinase inhibitors sorafenib and sunitinib induce cell growth inhibition in these cell lines. The effect of sorafenib resulted in the loss of active signaling molecules Erk1/2 in response to conditioned media in two of the three cell lines. We also show that sorafenib inhibits a multitude of signaling molecules in a cell line dependent manner but the loss of the pro-survival protein Mcl-1 was noted in all cell lines studied. We have also shown the synergistic activity of these agents with the topoisomerase I inhibitor irinotecan and provided evidence for the inhibition of NF-*κ*B activation as one potential advantage in this drug combination. We believe that the data presented here provide the basis for further studies to evaluate the effects of multi-tyrosine kinases in xenograft studies and subsequently for the formulation of clinical studies in patients with AT/RT.

## Methods

### Cell lines and cell culture

BT12 and BT16 cell lines were a gift from Drs. Peter Houghton and Jaclyn Biegel (St. Jude Children's Research Hospital, Memphis, TN, USA). These cell lines have been established from infants with CNS AT/RT (BT12 from a six-week-old female; BT16 from a two-year-old male). KCCF1 was established in our laboratory with cells obtained from the Cerebral Spinal Fluid (CSF) of a two-month-old male infant with AT/RT. Characterization of this cell line has been described previously [9].

Cells were cultured in Opti-MEM medium (Gibco, Invitrogen Corporation, Burlington, Ontario, Canada) supplemented with 5% fetal bovine serum (Gibco), 100 units/ml each of penicillin and streptomycin (Gibco). Confluent cells were trypsinized with 0.25% Trypsin-EDTA in Ca2+ and Mg2+ free balanced salt solution (Gibco) every three to five days. All cell cultures were maintained at 37 °C in a humidified atmosphere with 5% CO_2_.

### Multiplex cytokine assay

Cells (2 × 10^6^) in 2 ml of medium were grown for 48 hours and culture supernatants were analyzed for cytokine levels by multiplex technology as described previously [45]. Briefly, culture supernatants were diluted 1:4 in sample diluent buffer and mixed with beads containing capture antibodies. After incubation and washing steps, beads were mixed and incubated with biotin conjugated detection antibodies. Following the detection antibody incubation, each well was filter-washed and incubated in the dark with streptavidin-phycoerythrin (PE) conjugate. The plates were washed and the contents of each well were re-suspended in assay buffer. The plates were read in a Luminex100™ multiplex assay detection system (Luminex Corp., Austin, Texas, USA) and quantitatively analyzed using STarStation 2.0 software (Applied Cytometry, Sheffield, United Kingdom). In this study, a panel containing the following 65 human cytokines and chemokines was used: EGF, Eotaxin, FGF(basic), Flt-3, Ligand, Fractalkine, G-CSF, GM-CSF, GRO, IFN alpha2, IFN gamma, IL-1alpha, IL-1beta, IL-1Ra, IL-2, IL-3, IL-4, IL-5, IL-6, IL-7, IL-8, IL-9, IL-10, IL-12(p40), IL-12(p70), IL-13, IL-15, IL-17, IP-10, MCP-1, MCP-3, MDC, MIP-1alpha, MIP-1beta, PDGF-AA, PDGF-AB/BB, RANTES, CD40L, SDF-1, sIL-2R alpha, TGF alpha, TNFbeta, MCP-2, MCP-4, ENA-78, BCA-1, I-309, IL-16, MIP-1delta, TARC, 6Ckine, Eotaxin-2, Eotaxin-3, CTACK, IL-23, LIF, TPO, TRAIL, SCF, TSLIP, VEGF, IL-20, IL-21, IL-28A and IL-33.

### Antineoplasic agents

Sorafenib, sunitinib, irinotecan and SN-38 were obtained from ChemieTek (Indianapolis, Indiana, USA) and the Oncology pharmacy at the Alberta Children's Hospital. These agents were dissolved in DMSO (Sigma-Aldrich, Oakville, Ontario, Canada) to a final concentration of 10 mM and stored in aliquots at -20°C. At the time of study, agents were then appropriately diluted in culture medium.

### Cell growth inhibition assay

AT/RT cells were detached from the flask by trypsinization and plated in 96 well plates at a concentration of 1 × 10^3 ^to 5 × 10^3 ^cells per well. Increasing concentrations of study agents were added to these wells to a final volume of 200 µl per well. Corresponding dilutions of the vehicle DMSO was used as control. After four days in culture, cell survival was quantified by Alamar Blue Assay (Medicorp, Montreal, Quebec, Canada), according to manufacturer's protocol. Briefly, cells were incubated with 2.5% Alamar blue (which incorporates a proprietary redox indicator that changes color in response to metabolic activity) for 2 to 24 hours, and the absorbency at 570-620 nm was measured (Opsys MR Plate Reader, Dynex Technologies, Chantilly, Virginia, USA). Percent cell survival was calculated by:% Survival = (Abs 570-620 test well/Abs 570-620 Control) × 100.

From these values, inhibitory concentrations inducing 50% cell death (IC_50_) compared to DMSO wells were calculated. For drug combination studies irinotecan at IC_25 _concentration was added to cultures containing increasing concentrations of sorafenib or sunitinib. IC_50 _values were then calculated for these agents alone or in combination with irinotecan and used to derive Combination Index (CI) values as described previously [16]. A CI of less than 1 indicates synergy between the two agents under the experimental conditions used.

### Western Blot Analysis

In order to determine the expression of cellular targets of sorafenib and sunitinib in AT/RT cell lines, cells were grown to confluence in six well culture plates (Nunc, Rochester, New York, USA) over a 24 hour period. The media was removed and cells were washed with ice cold PBS and lysed in buffer containing 50 mM Tris, 5 mM EDTA, 0.1% SDS, 1% Triton X-100, 0.5% sodium deoxycholate with phosphatase and protease inhibitors (Sigma). Protein content of the lysates was measured by BCA Protein Assay Kit (Pierce, Rockford, Illinois, USA). Proteins (30µg/sample) were separated on an 8% polyacrylamide gel electrophoresis and transferred onto nitrocellulose membranes (Bio-Rad, Mississauga, Ontario, Canada). The membranes were blocked for two hours at 4°C with 5% skim milk powder in PBS containing 0.1% Tween-20 (Sigma). The blots were incubated with primary antibodies to c-Kit (1:1000, Santa Cruz Biotechnology, Santa Cruz, California, USA), PDGFR-β (1:1000, Santa Cruz), VEGFR2 (1:1000, Millipore, Billerica, Massachusetts, USA), Flt-3 (1:1000, Santa Cruz), c-Raf (1:1000, Cell Signaling Technology, Danvers, Massachusetts, USA), p-38α (1:1000, Santa Cruz) and β-actin (1: 10000, Sigma). After incubation overnight at 4°C, membranes were washed and probed with appropriate secondary antibodies conjugated to horseradish peroxidase (HRPO) (Sigma), followed by a luminal based substrate (Mandel, Guelph, Ontario, Canada) and developed by exposure to x-ray film (Fisher Scientific, Ottawa, Ontario, Canada). For intracellular signaling studies, cells were grown to confluence in six well culture plates and culture supernatant (spent medium) was removed, filtered and stored at 4°C and fresh serum free medium containing 10 μM sorafenib or vehicle (DMSO) control was added to the cells. After an additional two hours in culture the spent medium was added (10% v/v). Following further 30 min in the incubator, cells were lysed as described above and analyzed by Western blot using primary antibodies to Erk1/2 (1:1000, Cell Signaling), phospho-Erk1/2 (1:2000, R & D Systems, Minneapolis, Minnesota, USA), Akt1/2 (1:1000, Santa Cruz), phospho-Akt1/2 (1:1000, Santa Cruz), c-Raf (1:1000, Cell Signaling Technology), phospho-c-Raf (1:1000, Cell Signaling), Stat3 (1:1000, Cell Signaling), phospho-Stat3 (1:1000, Cell Signaling), Mcl-1 (1:1000, Santa Cruz) and β-actin. For the analysis of cytoplasmic NF-*κ*B, phospho-NF-*κ*B and I*κ*Bα, cells were grown in culture for 2 days, after which the media was removed and replaced with serum free media. The cells were then treated with sorafenib (10 µM) or DMSO control for 30 minutes, then irinotecan (10 µM) for an additional 2 hours. Cell lysates were then analyzed by using primary antibodies to NF-*κ*Bp65 (1:1000, Santa Cruz), phospho-NF*κ*Bp65 (1:1000, Cell Signaling), NF-*κ*Bp50 (1:2000, Millipore), I*κ*Bα (1:1000, Cell Signaling) and β-actin. For analysis of p27Kip1 expression, cells were treated with either sorafenib (1 µM) or irinotecan (1 µM) or both for 48 hours. Expression of p27Kip1 was determined using anti-p27Kip1 (1:1000, Cell Signaling).

### Immunofluorescence analysis of cytoplasmic NF-κB

BT12 cells were cultured in six well plates (2 × 10^6^/well) overnight and treated with (1) vehicle alone, (2) sorafenib (10 μM), (3) irinotecan (10 μM) and (4) sorafenib for 30 minutes followed by treatment with irinotecan for an additional 2 hours. Indirect immunofluoresence studies were carried out as described previously [46]. Briefly, after various treatments, cells were washed with cold PBS, fixed and incubated with antibodies to NF-κB (Santa Cruz) for 1 hour, followed by fluorescent labeled secondary antibodies (30 min). Concurrent DAPI staining was performed to locate nuclei in each slide. Slides were visualized under a fluorescent microscope and random fields were photographed.

## Competing interests

The authors declare that they have no competing interests.

## Authors' contributions

AJ planned and performed drug combination studies and Western blot analysis. DB planned and performed cytotoxicity studies. PB and KR facilitated the indirect immunofluorescence analysis and helped to write the manuscript. AN planned and supervised the studies and wrote the manuscript. All authors have read and approved the final manuscript.
